# Ancient deep-sea sponge grounds on the Flemish Cap and Grand Bank, northwest Atlantic

**DOI:** 10.1007/s00227-016-2839-5

**Published:** 2016-02-29

**Authors:** F. J. Murillo, E. Kenchington, J. M. Lawson, G. Li, D. J. W. Piper

**Affiliations:** Ecosystem Research Division, Department of Fisheries and Oceans, Bedford Institute of Oceanography, Dartmouth, NS B2Y 4A2 Canada; Natural Resources Canada, Geological Survey of Canada, Bedford Institute of Oceanography, Dartmouth, NS B2Y 4A2 Canada; Key Laboratory of Marginal Sea Geology, South China Sea Institute of Oceanology, Chinese Academy of Sciences, Guangzhou, 510300 China

## Abstract

Recent studies on deep-sea sponges have focused on mapping contemporary distributions while little work has been done to map historical distributions; historical distributions can provide valuable information on the time frame over which species have co-evolved and may provide insight into the reasons for their persistence or decline. Members of the sponge family Geodiidae are dominant members of deep-sea sponge assemblages in the northwestern Atlantic. They possess unique spicules called sterrasters, which undergo little transport in sediment and can therefore indicate the Geodiidae sponge historical presence when found in sediment cores. This study focuses on the slopes of Flemish Cap and Grand Bank, important fishing grounds off the coast of Newfoundland, Canada, in international waters. Sediment cores collected in 2009 and 2010 were visually inspected for sponge spicules. Cores containing spicules were sub-sampled and examined under a light microscope for the presence of sterrasters. These cores were also dated using X-radiographs and grouped into five time categories based on known sediment horizons, ranging from 17,000 years BP to the present. Chronological groupings identified Geodiidae sponges in four persistent sponge grounds. The oldest sterrasters were concentrated in the eastern region of the Flemish Cap and on the southeastern slope of the Grand Bank. Opportunistic sampling of a long core in the southeastern region of the Flemish Cap showed the continuous presence of sponge spicules to more than 130 ka BP. Our results indicate that the geodiids underwent a significant range expansion following deglaciation, and support a contemporary distribution that is not shaped by recent fishing activity.

## Introduction

Sponges (Phylum Porifera) are an ancient group of sessile animals present by the late Cryogenian 635 Ma (Maloof et al. 2010). There are four classes with recent species (Gazave et al. 2012): Calcarea (calcareous sponges), Hexactinellida (glass sponges), Demospongiae (siliceous sponges) and Homoscleromorpha (formerly included in Demospongiae), all well established in the Ordovician (480 Ma) when they may have played an important role in the evolution of eukaryotes, phosphorus removal and ocean oxygenation (Lenton et al. 2014). Despite their simple body plan, they are highly diverse, with approximately 8000 extant species described and potentially a further 7000 undescribed (Hooper et al. 2002). The vast majority of sponges are marine, where they occur in all oceans and to depths of 8840 m (Koltun 1970).

Sponge grounds in the northwest Atlantic are found along the continental slopes of the Grand Bank and Flemish Cap and northward along the Labrador Slope to the southern Davis Strait (Murillo et al. 2012; Knudby et al. 2013; Kenchington et al. 2014). Murillo et al. (2012) described four areas with large aggregations of sponges in the high seas east of Newfoundland, Canada, from an analysis of research vessel trawl catches. Sponge aggregations were found along: (1) the continental slope of the southeastern Grand Bank; (2) the southeastern slope of the Flemish Cap; (3) the eastern slope of the Flemish Cap; and (4) the northern slope of the Flemish Cap and the Flemish Pass in an area known as Sackville Spur. Faunal analyses of the benthic communities in this region have shown that these sponges form part of a distinct deep-sea sponge assemblage associated with sandy silt and clayed-silt bottoms with a high mud fraction, and are typified by high biomass of large sponges and high species richness (Murillo et al. 2015). Detailed in situ camera surveys extending beyond the trawl-sampled maximum depth on Sackville Spur showed that the sponge grounds there persist to depths of ~1700 m (Beazley et al. 2015). Kenchington et al. (2014) applying kernel density estimation to the research vessel trawl survey data used in Murillo et al. (2012, 2015), updated with new years and Canadian records, obtained high sponge biomass surfaces in the same areas previously identified and in one additional area on the southwest of Flemish Cap, east of Beothuk Knoll. Most of those areas of high biomass are currently protected from the damaging effects of bottom-contact fishing gear by the Northwest Atlantic Fisheries Organization (NAFO 2014) in accordance with the United Nations General Assembly Resolution 61/105, as sponge grounds have been identified as examples of vulnerable marine ecosystems (sensu FAO 2009).

Sponges constitute approximately 95 % of the total benthic invertebrate biomass on the Flemish Cap and approximately 50 % on the southeastern Grand Bank. In these areas, the Demosponges *Geodia barretti*, *G. phlegraei*, *G. macandrewii* (Geodiidae), *Stryphnus fortis* (identified as *S. ponderosus*) and *Stelletta normani* (Ancorinidae) are the main structure-forming sponges constituting more than 99 % of the total invertebrate biomass within the sponge grounds (Murillo et al. 2012) and reaching in some areas more than 3 mt/hectare. This northwest Atlantic sponge assemblage is very similar to that described by Klitgaard (1995) in the northeast Atlantic, where it is referred to as boreal “ostur”, occurring around the Faroe Islands, Norway, Sweden, parts of the western Barents Sea and south of Iceland where minimum water temperature is above 3 °C (Klitgaard and Tendal 2004).

**Fig. 1:**
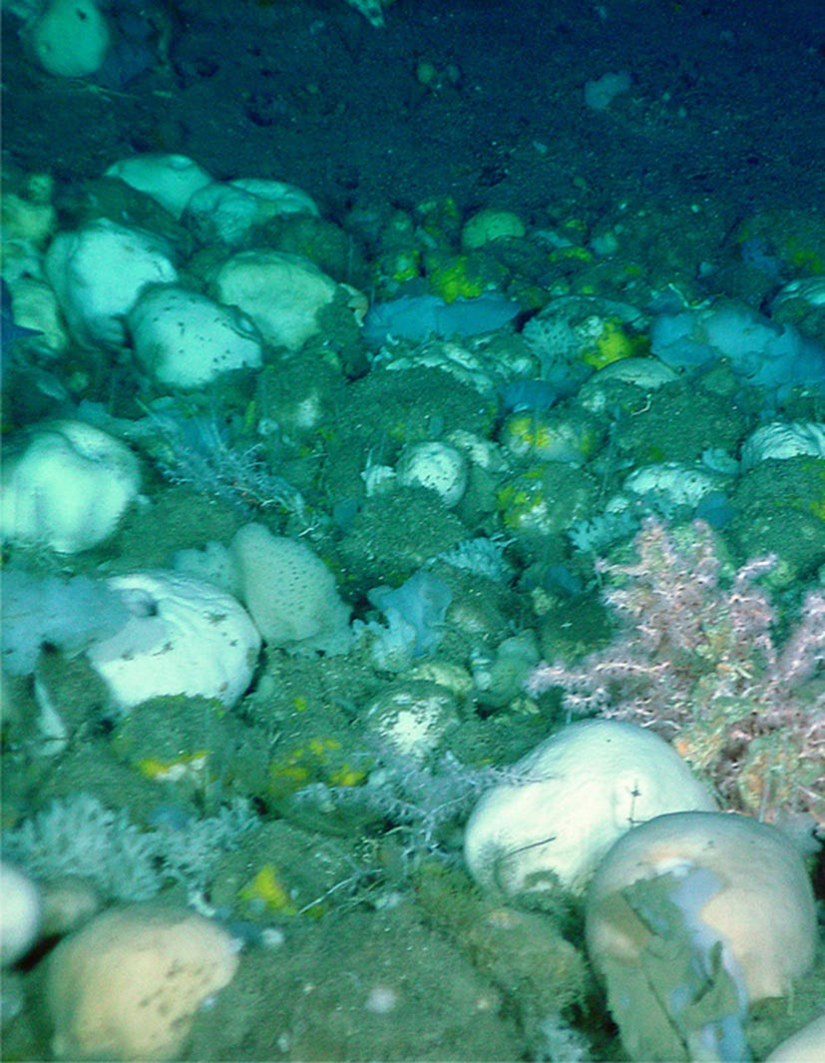
Extant sponge grounds dominated by species of *Geodia* on the northeastern slope of Flemish Cap at 1580 m depth. Bamboo corals (F. Isididae) can also be observed in the image

The sponge grounds in the Flemish Cap and southeastern Grand Bank region (Fig. 1) have sharp upper (shallow) depth boundaries (NAFO 2010; Murillo et al. 2012; Beazley et al. 2015) ranging from 800 m on the eastern slope of the Grand Bank to approximately 1300 m on Sackville Spur on the northern slope of Flemish Cap (Murillo et al. 2012; Barrio Froján et al. 2012; Beazley et al. 2015). Given the long history of fishing in the area (NAFO 2009) and the fact that sponges are easily dislodged by bottom-contact fishing gear (ICES 2009), it is possible that trawling has shaped these upper distributions. In that case, sponge grounds may have been much more extensive on Flemish Cap in the recent past and the current area closures may not be sufficient to restore habitat to a pre-disturbance state. However, as noted by Barrio Froján et al. (2012), it has not been possible to say for certain whether fishing is the direct cause behind this distributional pattern. Here, we present new information and a novel approach to address this issue that draws on the analysis of the presence of sponge spicules in the sediments surrounding the sponge grounds in the Flemish Cap and southeastern Grand Bank area.

Although the Demosponge grounds in the Flemish Cap and southeastern Grand Bank region do not form the dense spicule mats associated with the fused spicules of the Hexactinellid sponges (e.g. Dayton et al. 1974), they do accumulate upon the death of the sponge in significant enough quantities to form distinctive benthic habitats and communities (Barrio Froján et al. 2012). Sponge spicules have been shown to undergo little to no transport over several kilometres (Inoue 1985) and therefore are considered reliable indicators of the current and historical sponge presence or absence. Further, members of the Geodiidae can be easily distinguished from other Demosponge families because they uniquely possess microscleres called sterrasters found in the outer crust of the sponge (Uriz 2002). Fossils of Geodiidae date to the Early Cambrian, making it one of the earliest known Demosponge families (Reitner and Mehl 1995; Reitner and Worheide 2002). Therefore, the ancient history of the Geodiidae sponges in this area can be traced through the presence of sterrasters in sediment cores, permitting not only an evaluation of the persistence of sponge grounds in this area over recent and evolutionary time scales but also of the more specific time frame of the persistence of *Geodia*-dominated sponge grounds or “ostur” to evaluate the potential for co-adaptation of species.

## Materials and methods

### Study area: the Flemish Cap, Flemish Pass and slope of the Grand Bank

**Fig. 2:**
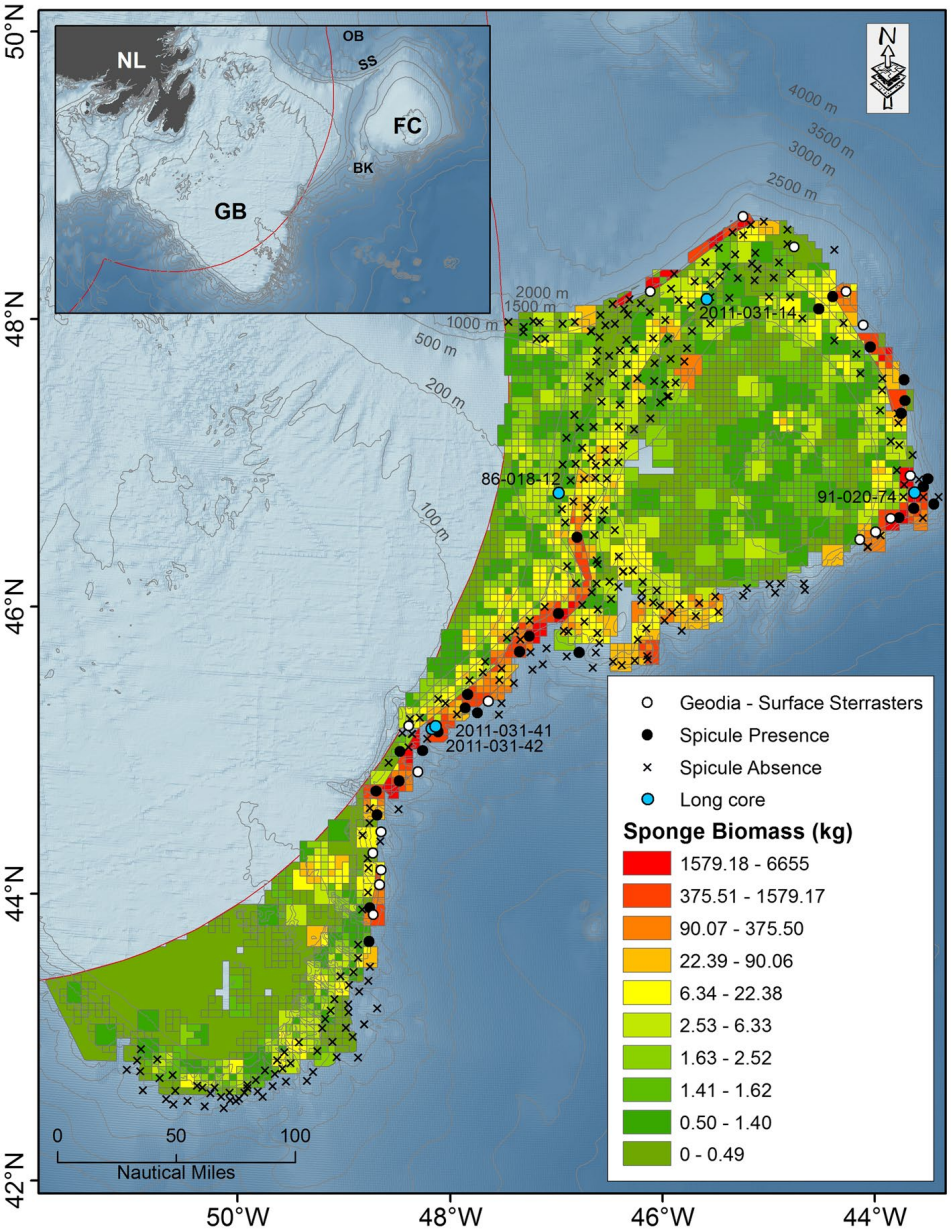
Location of Flemish Cap (FC) and Grand Bank (GB) off Newfoundland (NL) (see *inset*), comparing the location of samples with sponge spicules (*black solid circle*; *N* = 28), those with *Geodia* sterrasters in the surface layer (*white solid circle*; *N* = 17) and those without spicules (*black cross*) to the location of high sponge biomass areas identified by Spanish/EU research vessel trawl surveys (*grid squares* are 5 km × 5 km) recording sponge catch (kg). Outer edge of the trawl survey area is approximately 1500 m. The additional long cores examined are also indicated (*blue solid circle*; *N* = 5). The Canadian exclusive economic zone (EEZ) is indicated by a *red line*. *OB* Orphan Basin, *SS* Sackville Spur, *BK* Beothuk Knoll

The study area spans a portion of the continental margin offshore of eastern Canada comprising the Grand Bank slope, Flemish Pass and Flemish Cap. Grand Bank is a large submerged bank south and east of the island of Newfoundland. The continental slopes southeast and east of Grand Bank lead to the Newfoundland Basin and Flemish Pass, respectively. The Flemish Cap is an isolated offshore bank east of Grand Bank off Newfoundland (Fig. 2) that is underlain by continental crust of the Avalon terrane of the northern Appalachians (King et al. 1985). Approximately 118 Ma it lay close to North Africa, Europe and Greenland and has reached its current position through a combination of ocean spreading and crustal extension (Sibuet et al. 2007). It is separated from the Grand Bank to the west by the Flemish Pass, a channel that is approximately 1200 m deep.

Today, this area is swept by two predominant water currents: the southward-flowing Labrador Current and the northward-flowing North Atlantic Current. On the northern Grand Banks, the Labrador Current divides into two branches. The inshore branch flows southward close to the Newfoundland coast and carries about 15 % of the transport; and the warmer, deeper and faster offshore branch follows the shelf break around the Grand Bank carrying 85 % of the transport (Lazier and Wright 1993). When the current reaches the Flemish Cap region, the offshore branch subdivides into a strong southward branch flowing through the Flemish Pass to the southern slope of the Grand Bank and the eastward branch that circulates clock-wise around Flemish Cap. Around the Tail of the Grand Bank, the Labrador Current meets the Gulf Stream giving rise to the North Atlantic Current and its front. The North Atlantic Current flows around the Flemish Cap in the south and east, and below 300 m depth shows strong convergence near 45ºN with the southward-flowing Labrador Current (Gil et al. 2004). Strong temporal meandering of the North Atlantic Current explains the large temperature variability observed in this region (Lozier et al. 1995).

The last glacial maximum (LGM) in this region occurred between 28 and 20.5 ka BP (Shaw et al. 2006; Tripsanas and Piper 2008a) (All ages in this paper based on radiocarbon dating are given as calibrated ages, i.e. calendar years before 1950. The older literature may report ages in radiocarbon years: such ages have been recalibrated). At that time, glaciers extended to the edge of the continental shelves in most areas. However, the continental margins of Grand Bank and all of Flemish Cap were not glaciated, with the former above sea level and the latter below at the LGM (Shaw 2006). By 10 ka BP most of the Grand Bank was submerged (Shaw 2006). Deglaciation likely proceeded through ice calving at the margins which produced iceberg pitting and scouring on the sea bed, both processes continuing to the present day with iceberg rafting from higher latitudes (King et al. 1985). Iceberg scour extended to at least 650 m water depth around the LGM (Piper and Pereira 1992), but modern scours are principally in water depths of <250 m (Campbell et al. 2014).

The surficial geology of the study area is a product of modern oceanographic processes and past glacial activity (e.g. Piper and Pereira 1992; Sonnichsen and King 2005; Marshall et al. 2014; Weitzman et al. 2014). The surficial geology is variable, but in general, in water depths less than 600 m, the shallow geology consists of glacial till with a veneer of sand and gravel up to several metres thick. Bedrock is exposed at some locations. In deeper water, such as the slope of Grand Bank and Flemish Pass, the seabed generally consists of Holocene silty mud. On the steep upper slope off eastern and southern Flemish Cap and on parts of the floor of Flemish Pass, winnowed sands are present. On the extreme southeastern tip of Flemish Cap, calcareous ooze is accumulating.

### Field data collection

Contemporary distribution of sponge grounds in the study area was determined from sponge records obtained during groundfish bottom trawl surveys carried out for the assessment of fish stocks by Spain/Portugal of the European Union and Canada, from 1995 to 2013, with most data collected after 2002 (Kenchington et al. 2014). All surveys followed a depth-stratified random sampling design optimized for the target species with vessel speeds of approximately three knots. The data were drawn from three different combinations of gear type and trawling duration. In order to use all the sponge catches, we combined only the catches over 0.5 kg, where no significant differences between gear type or trawl duration were found (Kenchington et al. 2014). A sponge biomass surface was generated from those data in ArcGIS version 10.0 (ESRI 2011) following the methodology described in Cogswell et al. (2011). Additional records of geodiid species were taken from rock dredges and box cores collected through NEREIDA (http://www.nafo.int/science/nereida.html), a large-scale international habitat mapping programme operating within the study area. These complimented the sponge biomass surface produced from the research vessel trawl data, as these additional sampling tools were able to target areas not covered by the surveys.

Sediment samples (*N* = 339) were collected between 2009 and 2010 aboard the Spanish research vessel *Miguel Oliver* as part of NEREIDA. Samples targeting benthic infauna were taken using an ULSNER iron hot dip Mega Box Corer measuring 50 × 50 × 50 cm (sampled area of 0.25 m^2^). The box corer samples were sub-sampled using a 10-cm-diameter PVC tube (push core). Push cores were pressed into the sample, and a vacuum was maintained to extract the sediment. Push core tubes were sealed and refrigerated to prevent disruption of sediment during storage and transport. Locations of the samples are shown in Fig. 2.

In addition, a number of long cores (up to 6.5 m) collected by the Geological Survey of Canada from the study area were examined. These were not systematically reviewed for the sponge spicule presence during their processing; however, sponge spicules were noted in five of them, and those cores were included in our assessment (blue solid circle, Fig. 2) although only four of the five were sampled for geodiid sterrasters.

### Sediment core analysis

The push cores were brought to the Bedford Institute of Oceanography, Dartmouth, Nova Scotia, where they were divided in half and visually inspected for the presence of sponge spicules. Each push core subsample containing sponge spicules (*N* = 45) was stored in ethanol until processing in May and June 2011 and December 2013.

Various physical properties were measured on the split half cores: colour by spectrophotometry expressed in L* a* b* parameters (L* = lightness, 0–100 %; a* = green to red axis rating; b* = blue to yellow axis rating), magnetic susceptibility, bulk density and shear strength. All cores were digitally photographed and X-radiographed. Sediment types, such as sand and mud, were logged (Weitzman et al. 2014). Foraminiferan tests (shells) were separated from selected 5-cm-long samples from push cores and were dated by AMS radiocarbon techniques. Radiocarbon dates were calibrated using CALIB 6 with Δ*R* = 144 ± 38 years (Weitzman et al. 2014).

Correlations were made between adjacent cores to develop a regional lithostratigraphy. This correlation used in particular sediment colour, since L* (black to white) is a proxy for carbonate content and the abundance of ice-rafted gravel observed in X-radiographs, which commonly corresponds to high magnetic susceptibility (Weitzman et al. 2014).

To determine the reliability of spicules in sediment cores as a predictor of the presence or absence of sponge grounds, data from the surface of the sediment core (0–1 cm) were compared to the known presence of sponges in the area as sampled by the research trawlers and from rock dredges and box cores collected through NEREIDA.

### Sterraster identification and quantification

In the laboratory, forceps were used to collect three sub-samples from each region of the push core with spicule deposition. Glass slides were weighed before placing the sub-sample on the slide. The sub-sample was dissolved in water to help separate the sediment from the spicules. The sub-sample was placed under a light microscope, and megaspicules were photographed at 4× magnification. At 10× magnification, sterrasters were counted using a click counter. Quantities of sterrasters per sub-sample were recorded, and the slide was dried before weighing. The numbers of sterrasters per sub-sample were standardized to the weight of the sample, so that the average sterraster abundance was determined per 0.01 g of sediment examined. Lastly, a glass coverslip was placed over top of a random section of the slide. Photographs were taken at 20× magnification and 40× magnification as needed. In the few cases where there were few or no spicules, the slide was only viewed and photographed at 4× and 10× magnifications. Therefore, all samples with sterrasters also had other spicules present.

## Results

### Core stratigraphy and chronology

Most push cores were 30–60 cm in length. All of the push cores were bioturbated at the surface, and in some cases the sediments may have been additionally slightly disturbed by the coring process, although any cores that were highly disturbed so as to invalidate the findings were not processed. Five sediment units, A to E from top to bottom, were recognized (Table 1). Most cores penetrated only units A and B. Units D and E were found in only a few cores that showed erosional hiatuses.

**Table 1:** Summary of lithostratigraphic units

Unit	Sub-unit	Characteristics
A		Olive brown to olive grey sediment at top of core, sparse IRD
	A	Mud or silty mud
	A′	Sand or muddy sand
B		Mud with common IRD, variably olive brown to light brown, bioturbated
C		Dark grey to grey brown mud with sparse IRD, lower L* than in B
D		Very light brown mud, orange hue. Heinrich^a^ layer 1
E		Dark olive brown or grey mud, some IRD, underlies unit D

*IRD* coarse-grained (>1 mm) ice-rafted detritus, L* lightness colour parameter

^a^Heinrich layers are distinctive North Atlantic sediment layers that record well-dated episodes of major iceberg release

**Fig. 3:**
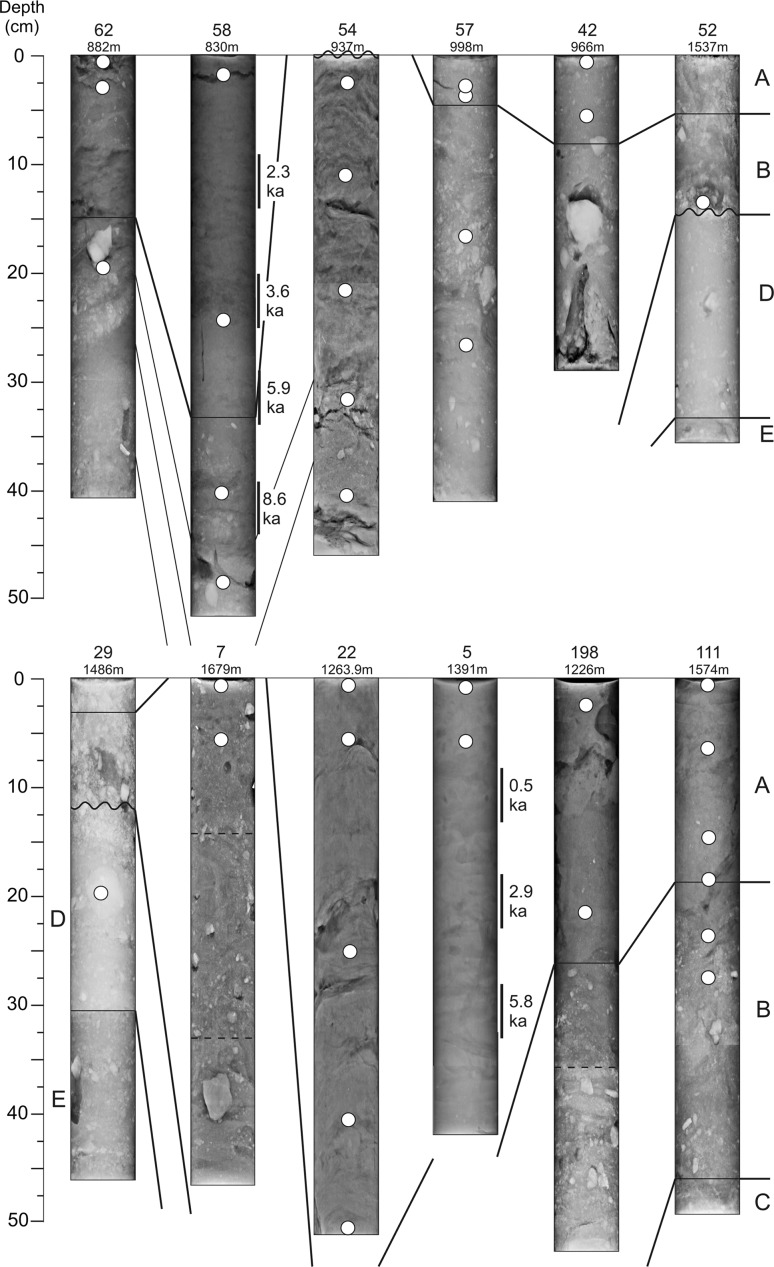
Example of push cores and lithostratigraphic units (see Table 1 for definitions). Depth of sponge spicules is shown by *white dots*. Radiocarbon dates in calendar years BP were based on bulk foraminiferan tests from 5-cm sediment intervals. The *number* of the push core and the depth at which it was collected are indicated above each core

Much of the sediment supply to the flanks of Flemish Cap is transported in the Labrador Current. As a result, sedimentary units can be correlated with those found farther north beneath the Labrador Current in Orphan Basin (Tripsanas and Piper 2008b) and on the Labrador Shelf (Andrews et al. 1999). Unit A generally comprises muds with low carbonate content, locally represented by winnowed sand or by calcareous biogenic ooze. Several radiocarbon dates confirm that it is younger than 6.5 ka and correlates with the Maqqak Clay unit on the Labrador Shelf (Josenhans et al. 1986). Unit B is defined by the onset of common ice-rafted gravel (visible as bright spots in X-radiographs, Fig. 3), but also generally has a higher carbonate content than unit A, represented by a lighter tan colour with higher L* colour values and appearing brighter in X-radiographs. Unit B corresponds to the widespread early Holocene supply of detrital carbonate through Hudson Strait to the Labrador Current. Based on correlation with core 2003-033-24 in southern Orphan Basin (Tripsanas and Piper 2008b), the lowest carbonate-rich interval in unit B probably corresponds to the end of the Younger Dryas (~11.5 ka). The most prominent Holocene carbonate-rich layer in Orphan Basin corresponds to the Gold Cove event at ~11.2 ka, but radiocarbon dates suggest that prominent carbonate-rich layers in some cores may represent events as young as the Foxe Basin event at 6.6 ka. Unit B thus corresponds to the Qeovik Silt unit of the Labrador Shelf. Unit C is a darker grey silty mud with lower carbonate content than either the overlying unit B or the underlying unit D. Unit D is identified as the very distinctive Heinrich layer 1 (~17.5–16.7 ka), based on the presence of common ice-rafted gravel and high spectrophotometer values of both a* (red colour) and L* (carbonate) in comparison with well-dated cores in Orphan Basin (Tripsanas and Piper 2008b) and Flemish Pass (Huppertz and Piper 2009). Unit E is a grey mud with ice-rafted gravel underlying unit D.

### Chronological distribution of geodiid sterrasters and other sponge spicules

**Fig. 4:**
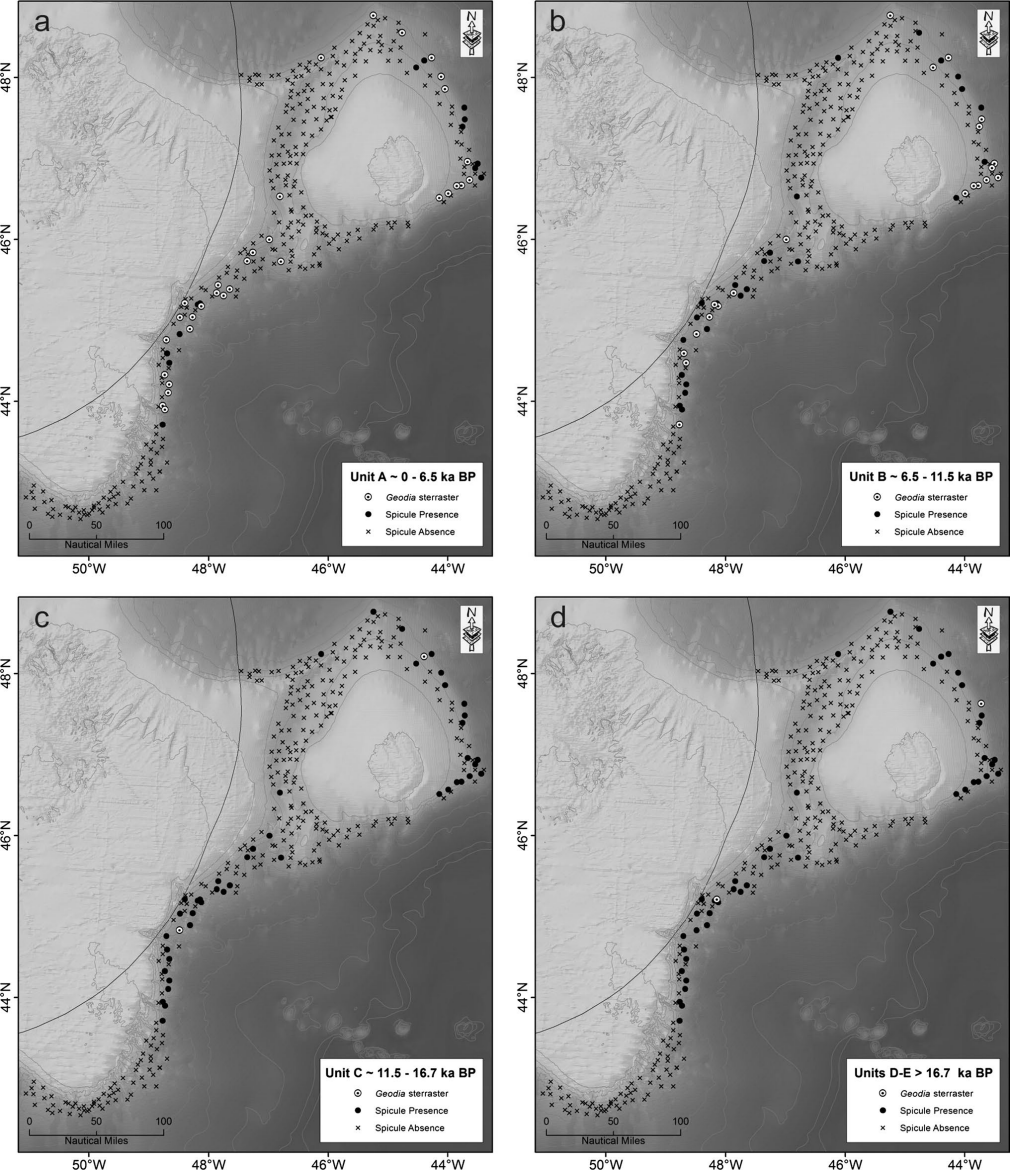
Map of the Flemish Cap and Grand Bank slope, comparing the presence (*filled circle*) or absence (*cross*) of sponge spicules and *Geodia* sterrasters (*open large circles* with *dark centres*) in sediment cores among the chronological groupings. **a** Unit A ~0–6.5 ka. **b** Unit B ~6.5–11.5 ka. **c** Unit C ~11.5–16.7 ka. **d** Units D and E >16.7 ka

The megascleres seen in the cores were typical of the order Tetractinellida but were not identified further. The presence or absence of geodiid sterrasters in sediment cores was compared among the five chronological groupings (present at the seabed surface; unit A ~0–6.5 ka; unit B ~6.5–11.5 ka; unit C ~11.5–16.7 ka; unit D ~16.7–17.5 ka; unit E >17.5 ka, Fig. 4). Chronological groupings showed a widespread, yet predictable distribution of sterrasters, remaining confined to most of the regions where contemporary high sponge biomass (Figs. 1, 2, and Kenchington et al. 2014) was found (Fig. 4; panels 1–4): the continental slope of the Grand Bank; E-SE Flemish Cap; and Sackville Spur (N Flemish Pass). No sterrasters were found on the southwest of the Flemish Cap east of Beothuk Knoll despite a high contemporary biomass of sponge in the vicinity of the cored areas (Fig. 2). The most recent chronological grouping (Fig. 4, panel 1) showed the widest distribution of sterrasters, but also included the greatest sample size. Sterrasters in the oldest chronological grouping (~11,500 years BP and older) from our push core data were confined to the east of the Flemish Cap and southeastern slope of the Grand Bank (Fig. 4; panels 3 and 4).

**Fig. 5:**
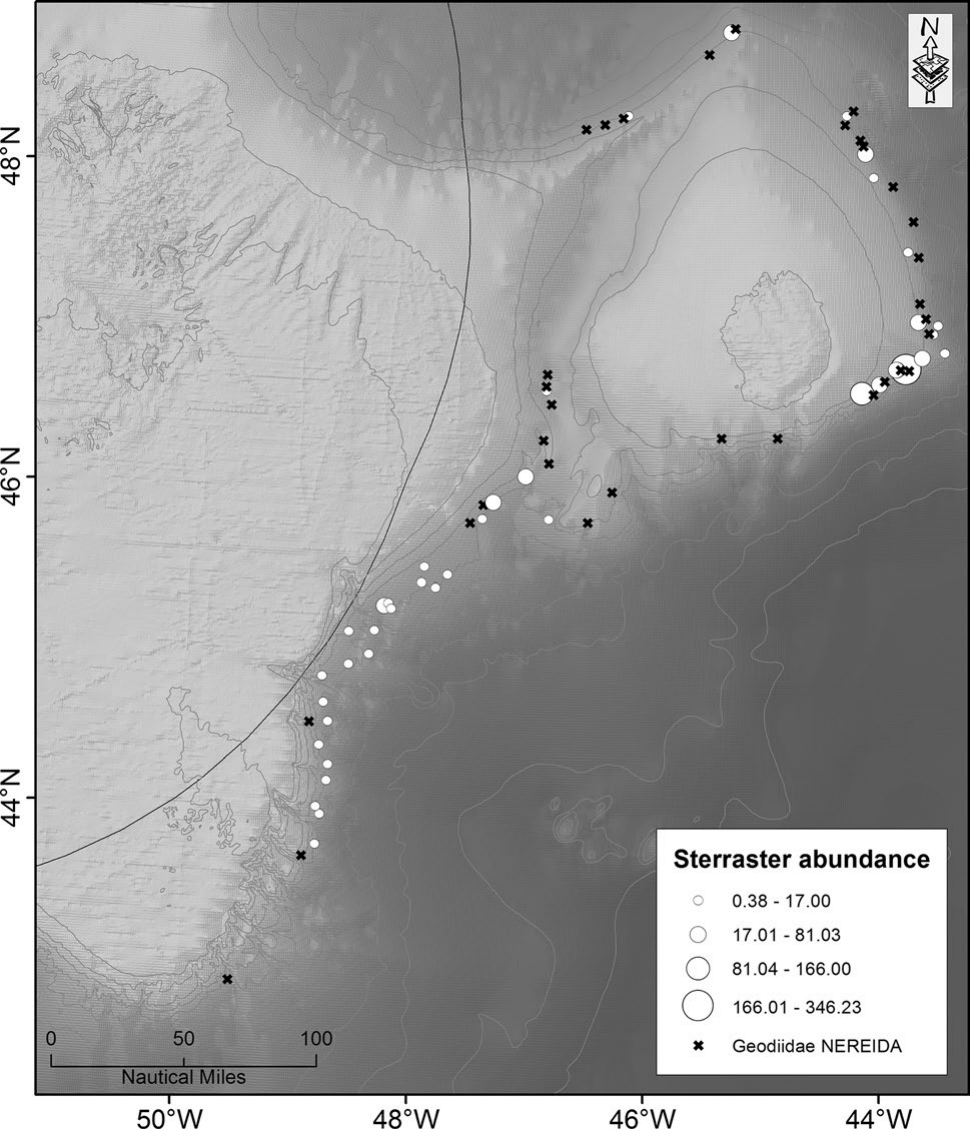
Sterraster abundance per 0.01 g of sediment and contemporary Geodiidae records collected from rock dredges and *box corer* through NEREIDA

Highest sterraster abundances were found in the southeastern tip of the Flemish Cap (Fig. 5), whereas lowest abundances by comparison were found in the northern half of the Flemish Cap and slope of the Grand Bank. Geodiidae records obtained through NEREIDA present a similar distribution pattern to that of the sterrasters and were also confined to the high sponge biomass areas (Fig. 5).

**Fig. 6:**
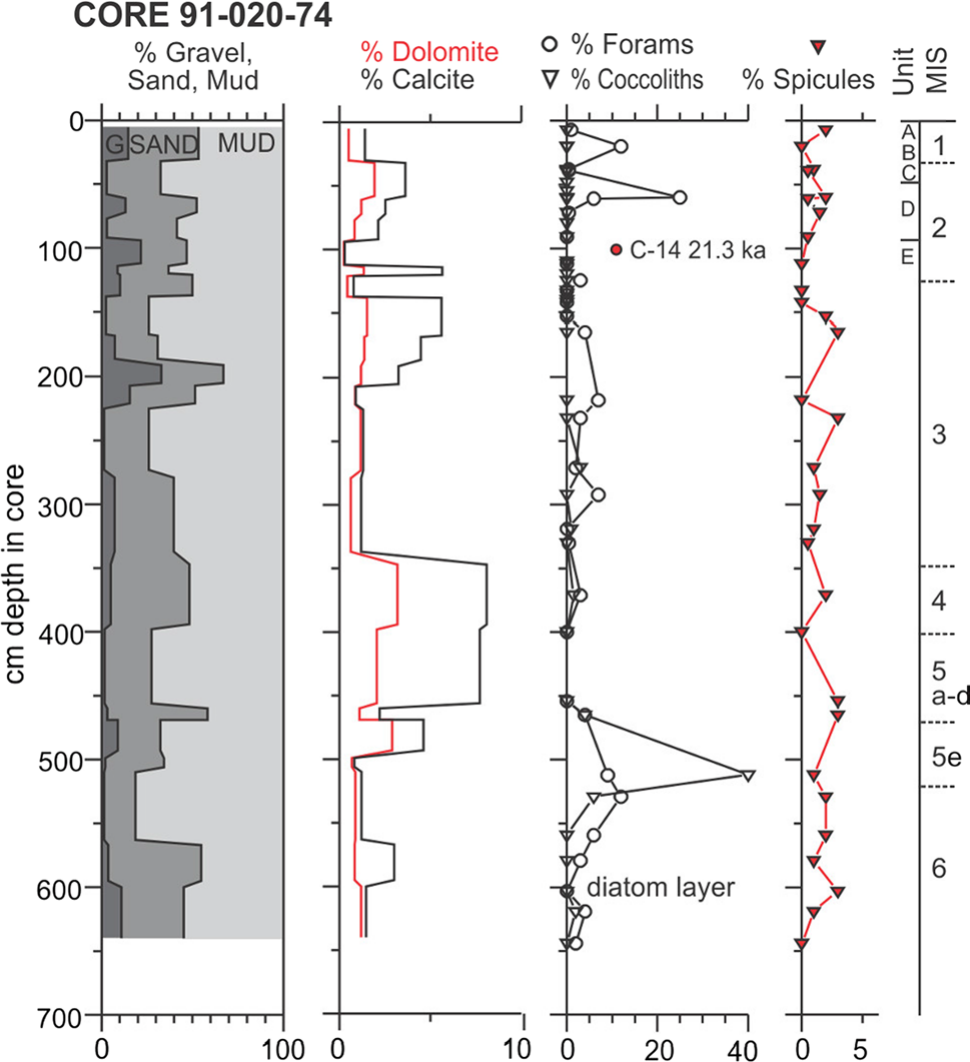
Presence of sponge spicules in a long core taken from the southeast of Flemish Cap (*right panel*) in relation to depth down the core, percentage of sand, carbonate and plankton frustules (forams, coccoliths and diatoms). MIS are marine isotopic stages, A–E are lithostratigraphic units in Fig. 3

Sterrasters were found in the two long cores on the slope of the Grand Bank. One of them, (Core 2011-031-42), was quite unusual. The sterrasters were found in the top of a submarine landslide deposit so their age is not well known, but it is certainly older than 25 ka (based on the character of the undisturbed overlying sediment) making this the oldest record for geodiids in this region. An entire geodiid sponge was recovered in Unit E of core 86-018-12 in western Flemish Pass in 933 m water depth (Piper and Pereira 1992). The long core (91-020-74, Fig. 6), collected at the southeast of Flemish cap in 972 m water depth (Fig. 2), showed sponge spicules distributed along the entire core, with the oldest from more than 130 ka BP. This is the oldest record and the oldest continuous record of sponges from the region.

## Discussion

This study has identified the presence of ancient geodiid sponge grounds occurring on the Flemish Cap and Grand Bank from ~17 ka to present. Sponge grounds were not detected at any period from the extreme southeastern Tail of Grand Bank and from the northern Flemish Pass between the Nose of Grand Bank and Flemish Cap. Contemporary Geodiidae tend to be found in constant environmental conditions, with stability in water mass characteristics, particle content and low disturbance regimes (Klitgaard and Tendal 2004; Beazley et al. 2015). However, the geological evidence suggests considerable change in the environmental conditions of this region over geological timescales, particularly on southeast Flemish Cap. The presence of abundant spicules in Heinrich Layer 1 in core 29 with an accumulation rate of ~0.2 mm/a (millimetres per year) contrasts with present conditions with a mean accumulation rate of 0.01 mm/a. Coccolith abundance in core 58 and also in 91-020-74 (Fig. 6) shows that the southeast Flemish Cap was bathed by warm North Atlantic Drift waters in the late Holocene and occasionally in the past 130 ka, but otherwise was influenced by Labrador Current water. Bottom waters may have been more stable through time, but there have surely been changes in near-surface productivity. Analysis of variability in Labrador Current strength, using grain-size variations from cores and sedimentation rates across a transect from Flemish Pass, provides evidence that the Labrador Current from at least 24 to 16 ka was relatively weaker and from 16 ka to present was stronger and increasing in strength (Marshall et al. 2014). This increase in bottom current speed could explain the significant range expansion of geodiids following deglaciation in the northwestern Atlantic region observed in this study. In support of this view, we noted that the sponge grounds found in this region are strongly associated with bottom current speed (Knudby et al. 2013) and sterrasters dated older than 11.5 ka were scarce, although we have confirmed from one of the long cores that they were present before the LGM.

We used our data to address the question of whether the upper depth distribution of the sponge grounds has been shaped by fishing removals over the last half century. The Greenland halibut fishery is the main fishery carried out in waters below 700 m depth in this area (González-Costas et al. 2011). The fishery began in the early 1960s off eastern Newfoundland and in 1990 was intensively developed in the deep water area of the north Flemish Cap (Sackville Spur) and Flemish Pass (Bowering and Brodie 1995) in close proximity to the current distribution of the sponge grounds. This fishery distribution has been quite constant since then (González-Troncoso et al. 2007; Campanis et al. 2008). Despite a close proximity, the fishing distribution does not overlap with areas of high sponge biomass (Murillo et al. 2012), although recording of sponge bycatch started several years later than the fishery. The species distribution models previously discussed (Knudby et al. 2013) predicted the probability of occurrence of sponge grounds in this area based on different environmental variables and showed excellent concordance with the location of the sponge concentrations identified from the survey data and low probability of the presence on the actual fishing grounds (Knudby et al. 2013). Our study supported those models. Neither sterrasters nor spicules were observed on the cores from the fishing grounds-suggesting environmental drivers, perhaps combined with intrinsic biological characteristics governing larval dispersal, likely explained the contemporary upper depth distribution of the geodiid sponge grounds. Therefore, the contemporary distribution of the sponges in the Flemish Cap area is likely a natural distribution and not one shaped by fishing activity to any great extent. Mapping of the historical distributions together with other techniques such as species distribution modelling can be a very useful approach for evaluating protection measures in fisheries management, particularly with respect to the restoration potential of closure boundaries.

Blacker (1957) identified *Geodia barretti*, a dominant geodiid in the northeastern Atlantic with temperature tolerance ranges from 1 to 6 °C and absent at water temperatures under 1 °C, as an excellent indicator species for Atlantic oceanographic conditions. Similarly, the geodiid species that constitute the sponge grounds of northwestern Atlantic are mainly boreal species (Murillo et al. 2012; Cárdenas et al. 2013) associated with the flow of the Labrador Current with temperatures between 3 and 4 °C (Colbourne and Foote 2000). This stable bottom temperature is favourable for geodiid development and is within its temperature tolerance, as defined by Blacker (1957). A detailed study from the Sackville Spur area has related the sponge grounds present in this area to a remnant of the Irminger Current that is warmer and saltier than expected for Labrador Sea Water and that could account for the upper and lower depth boundaries in this area (Beazley et al. 2015). However, direct observations should be made to confirm the presence of this Irminger remnant and on the ocean physics of other areas with sponge grounds.

Our historical analysis of geodiid distribution and abundance over time used sterrasters as an indicator of the presence or absence of previously living sponges. Previous investigations in Sagami Bay, Japan (Inoue 1985), concluded that sponge spicules in sediment underwent little or no transport over an area of several kilometres, although some types of spicules did not have an equivalent distribution between the sediment and the living sponge, as was the case of sterrasters and triaenes. Inoue (1985) treated sterrasters separately from other spicules because sterrasters having an ovoid form were considered to behave differently in transportation processes. Our comparison between surface sterrasters using both the research vessel survey data (Fig. 2) and the rock dredge and box corer geodiid collections (Fig. 5) confirmed that the presence of sterrasters in sediment cores provided a reliable indicator of the geodiid presence or absence, rather than being deposited a great distance away from living populations. The only anomaly occurs to the east of Beothuk Knoll where sponge biomass is currently high and spicules were absent from the push core, although cores were not taken from the peak biomass area. Further, if trawling had the effect of redistributing the spicules, we would have seen a different distribution between the surface observations and those pre-dating the trawling, and this was not the case. There would also be a lack of concordance between the species distribution models (Knudby et al. 2013) and the presence of spicules.

In conclusion, Geodiidae-dominated sponge grounds in the Flemish Cap and slope of the Grand Bank can be dated back to the end of the last glacial maximum, and their persistence and abundance provide valuable information on the influence of dominant water masses over a long period of time and for investigating adaptation. Our data are consistent with the contemporary spatial configuration of the sponge grounds being reflective of habitat occupancy over the past millennia. Sterraster absence in shallower sampled areas also indicates that these sponge grounds were not previously present in the currently fished areas, on the extreme southeastern slope of the Tail of Grand Bank, or on any of the shallower waters (at least to ~500 m) of Flemish Cap.
